# Validated prediction of xerostomia in a real-world population: a step toward model-guided radiotherapy

**DOI:** 10.2340/1651-226X.2025.43462

**Published:** 2025-08-18

**Authors:** Emmy Dalqvist, Tiziana Rancati, Anna Embring, Gabriella Alexandersson von Döbeln, Ingmar Lax, Signe Friesland, Eva Onjukka

**Affiliations:** a Department of Oncology Pathology, Karolinska Institutet, Stockholm, Sweden; b Department of Nuclear Medicine and Medical Physics, Karolinska University Hospital, Stockholm, Sweden; c Data Science Unit, Fondazione IRCCS Istituto Nazionale dei Tumori, Milan, Italy; d Department of Radiotherapy, Karolinska University Hospital, Stockholm, Sweden; e Department of Clinical Science, Intervention and Technology, Karolinska Institutet, Stockholm, Sweden; f Department of Head, Neck, Lung and Skin Tumors, Karolinska University Hospital, Stockholm, Sweden

**Keywords:** external validation, xerostomia, head and neck cancer, dose–response relationship, normal tissue complication probability, radiation therapy

## Abstract

**Background and purpose:**

The aim of this study is to validate an Normal Tissue Complication Probability (NTCP) model for xerostomia in a large quality-registry cohort, enabling its future use in individualized NTCP-based treatment planning.

**Material and methods:**

A model predicting grade ≥ 2 xerostomia (6 months post-radiotherapy) was selected for validation, including the mean dose to both the parotid and the submandibular glands, in addition to the baseline score for xerostomia, as predictors. Our local validation cohort consisted of 674 patients (204 events), treated between 2012 and 2024, with a median follow-up of 10.3 months (range 5–24). A closed testing procedure was performed to investigate the need for model updating, and the performance of the models was assessed with calibration curves, discrimination, the Brier score, and the Hosmer-Lemeshow test.

**Results:**

The calibration curve demonstrated that the model predicted the dose–response relationship well. The validation cohort showed a slightly stronger dose response, with a slope of 1.16. The calibration intercept of −0.12 revealed an overestimation of xerostomia.

However, the closed testing procedure indicated that a recalibration of the model was needed, and the HL-test showed a significant deviation. The recalibrated model showed perfect calibration but still limited discrimination (Area Under the Curve (AUC) 0.62).

**Conclusion:**

The validated model performed well in our real-life dataset despite the differences between the training and validation cohorts, particularly considering the lack of baseline score in our cohort. This highlights the potential for improved performance with baseline inclusion but still suggests that an individualized NTCP-based treatment-planning protocol can be developed using the recalibrated published model.

## Introduction

Xerostomia, or dry mouth, is a common and debilitating side effect of head and neck radiotherapy, resulting from radiation-induced damage to the salivary glands and significantly reducing patients’ quality of life [1]. The relationship between radiation dose and xerostomia has been extensively studied using Normal Tissue Complication Probability (NTCP) models [2–8]. Historically, these models have primarily focused on the mean dose to the parotid glands [3, 7, 8]. However, more recent studies have also incorporated the submandibular glands [9, 10] and the minor salivary glands within the oral cavity [2]. When modeling xerostomia, previous research has highlighted the importance of accounting for baseline patient characteristics, which is evaluated before treatment [2, 3]. Evidence suggests that non-dosimetric factors also influence the risk of xerostomia; for example, age and concomitant chemotherapy [3, 6] have been identified as important contributors. Technological advancements have further propelled progress in the field by incorporating radiomic features [11], spatial information [12, 13], and other features of the 3D dose-distribution [10, 14] to improve the predictive accuracy of NTCP models for xerostomia. While these developments significantly enhance our understanding of the multifactorial mechanisms underlying the occurrence of side effects on an individual basis, their integration into routine clinical workflows remains challenging. Despite these advancements, many clinical practices continue to rely on non-individualized dose constraints, such as point doses or dose-volume histogram (DVH) metrics, rather than adopting the NTCP-based dose reporting to organs at risk (OAR), as recommended by the International Commission on Radiation Units and Measurements (ICRU) [15]. Therefore, the validation of NTCP models that are more readily implementable in a clinical setting is the key to a more evidence-based treatment-planning approach.

The Transparent Reporting of a Multivariable Prediction Model for Individual Prognosis or Diagnosis (TRIPOD) statement [16] is widely used for reporting model development or evaluation, but it does not outline specific methodologies for these tasks. A systematic review from Sharabiani et al. [17] highlighted that only a limited number of studies on NTCP models for head and neck toxicities have been subject to external validation. External validation is necessary to assess a model’s reproducibility and generalizability, which are critical concerns when relying on the model for decision support in clinical practice. Many researchers have emphasized the importance of external validation [17, 18], and several methodological frameworks have been published in recent years [18–22]. The focus should now be placed on validation studies to prove the validity of preexisting models.

Van den Bosch et al. [2] have identified individual toxicity risk profiles for head and neck treatments and developed models for xerostomia and dysphagia at multiple time points after treatment. The national Dutch protocol by Langendijk et al. [23] refined these models through recalibration, using a combined cohort of 1145 patients, achieving the level of a TRIPOD type 4a model. This model is currently used in the Netherlands to allocate patients to proton or photon treatments, based on the estimated benefit [24]. In Sweden, patient selection for proton therapy is based on a consensus-driven approach. The aim of this validation is not to challenge the current national strategy but rather to pave the way for an alternative clinical application of NTCP models. Specifically, models may support personalized treatment planning by allowing dose prescription to be tailored according to individual anatomical characteristics and the associated personal risk of side effects.

Since 2014, acute and late side-effects after radiotherapy have been systematically recorded in a local quality registry for head and neck cancer at the Karolinska University Hospital. The current study aims to externally validate the NTCP model for grade ≥ 2 xerostomia from the national Dutch protocol within a suitable subgroup of this registry. The objective is to assess the generalizability of the model to our clinical patient cohort, with the potential future use of iso-NTCP prescription to optimize treatment outcomes while maintaining an acceptable toxicity profile.

## Material and methods

The model for xerostomia of grade ≥ 2 (6 months post-radiotherapy) from the Dutch National Indication Protocol for Proton Therapy [23] was selected for validation (hereafter referred to as the LIPP model).

### Patient selection

A validation cohort was extracted from the local quality registry for head and neck cancer at the Karolinska University Hospital. Details of the registry have been described previously by Onjukka et al. [4]. The inclusion criteria for the current cohort were as follows: treatment between 2012 and 2024 with a follow-up time between 5 months and 2 years; the parotid- and submandibular glands contoured as part of clinical routine; curative intent (i.e. prescribed dose ≥ 50 Gy); and tumor location in the proximity of the salivary glands, including cancer of the oral cavity, oropharynx, nasopharynx, hypopharynx, oral cavity, larynx, or other sites (nasal cavity, paranasal sinuses, or metastases in the neck with an unknown primary). Patients who received plan adaptation during the radiotherapy course were excluded due to the risk of anatomical changes leading to considerable uncertainty in the accumulated dose to the evaluated OAR. Similarly, patients who received reirradiation or a brachytherapy boost were also excluded. Dose-volume histograms (DVHs) for the parotid and the submandibular glands were extracted from the Eclipse treatment-planning system (Varian, USA) using a locally developed script.

### Treatment

All patients received high-dose radiotherapy with a curative intent, 50 to 84 Gy in 17 to 35 fractions, alone or with induction chemotherapy and/or concomitant medical therapy (chemotherapy or targeted therapy). The predominant treatment approach was the simultaneous integrated boost (SIB) technique (since 2016). In 2020, the consensus guidelines from Grégoire et al. [25] including the 5 + 5-mm clinical target volume (CTV) expansion were implemented, with 68 Gy in 34 fractions to the gross tumor volume (GTV), 61.2 Gy to CTV_68 + 5 mm, and 51.68 Gy to the elective volume. Previously, the 68 Gy CTV included the GTV with a 10-mm margin, while the elective CTV was unchanged (meaning only two prescription levels were used). The parotid and the submandibular glands were delineated based on their appearance in the CT image. Knowledge-based auto-planning (RapidPlan: Varian Medical Systems, Palo Alto, USA) was used from 2019.

### Xerostomia endpoint

The modeled toxicity endpoint was late xerostomia of grade ≥ 2 (moderate-to-severe xerostomia), assessed by clinicians according to the RTOG/EORTC Radiation Morbidity Scales [26]. As the registry consists of real-world data, the follow-up time is not consistently at 6 months post-radiotherapy, as for the LIPP cohort. The number of follow-up assessments registered for each patient varied across the cohort. Where multiple assessments were available, the follow-up nearest 6 months was selected but limited to a maximum of 2 years (as per the inclusion criteria). This study was approved by the regional ethics committee (2016/268-31/1).

### Statistical analysis

#### Reference model

Van den Bosch et al. developed a model on the prospective CITOR cohort with data from the University Medical Centre Groningen (UMCG) in the Netherlands (750), validated on data from UMCG, the Maastro Clinic, and the Radiotherapeutic Institute Friesland (RIF) (395) [2]. The latest version of the LIPP model (version 2.2) represents an updated and recalibrated version of the Van den Bosch model, developed using a combined cohort of 1145 patients [23]. Model refinement was conducted using a closed testing procedure [27]. In the combined cohort, the prevalence of the endpoint was 46%, assessed via the EORTC QLQ-H&N35 questionnaire. The model incorporates the mean dose to both the parotid (ipsilateral [ips] parotid and contralateral [con] parotid) and the submandibular (SMG) glands, in addition to the baseline score for xerostomia.

NTCP_LIPP_ = (1+e^-S^)^-1^

where:


$$S=-2.295+0.0996*\left(\sqrt{D_{mean}\left(ips\;Parotid\right)}+\sqrt{D_{mean}\left(con\;Parotid\right)}\right)+0.0182*\left(D_{mean}\left(SMG\right)\right)+Baseline\;score$$

The baseline score was 0 for none, 0.459 for mild, and 1.207 for moderate to severe xerostomia, at baseline.

#### Validation procedure

Possible differences between the current cohort and the LIPP cohort were evaluated using a chi-square test for categorical values and a t-test for continuous values, setting the significance threshold at *p* < 0.05. Specifically, the following patient and treatment characteristics were considered: sex, age, tumor site, human papillomavirus (HPV) status (for patients with oropharyngeal cancer), concomitant medical treatments, induction chemotherapy, radiotherapy schedule, radiotherapy technique, xerostomia rate, and the mean physical doses to the parotid and the submandibular glands.

In the validation cohort of the current study, baseline data were only available for a minority of the patients (around 15%), and a baseline score of zero was assumed for all patients. The closed testing procedure [27] was employed to evaluate the necessity for model updates, considering three potential approaches: calibration-in-the-large (re-estimation of the model intercept), recalibration (re-estimation of both the intercept and slope), and model revision (re-estimation of all model coefficients). Based on the results of the closed testing procedure, the appropriate updates were applied to optimize the model’s predictive performance and ensure its suitability for clinical application.

The model performance was assessed by calibration and discrimination metrics. A calibration curve was generated to visually compare the predicted versus observed probabilities of xerostomia grade ≥ 2. The calibration was evaluated using calibration-in-the-large, calibration intercept and slope, together with the Brier score and the Hosmer-Lemeshow (HL) goodness-of-fit test. The discrimination was tested using the area under the receiver operating characteristic curve (AUC). The clinical usefulness of the model was analyzed using the net benefit [28, 29] metric. A detailed description of this approach is provided in the Supplementary Materials.

MATLAB (version R2019a) was used for all statistical analyses, and all DVHs were converted to equivalent dose in 2 Gy per fraction (EQD2) using the linear-quadratic model, with α/β = 3 Gy.

## Results

### Cohort characteristics

**Figure 1:**
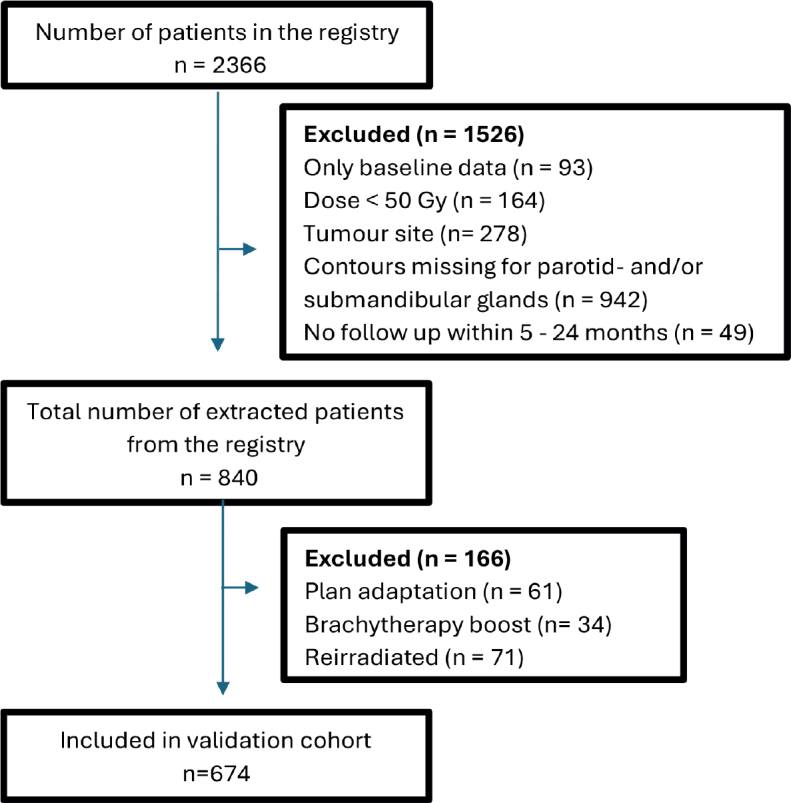
Flow chart of patient exclusion of the KUH cohort. The number of excluded patients prior to data extraction from the registry is presented in the order in which the exclusion criteria were applied, as overlaps between criteria may occur.

At the time of this analysis, the registry comprised 2366 patients. Out of these, 840 patients fulfilled the inclusion criteria. After excluding 61 patients who received plan adaptation, 34 patients who received brachy boost, and 71 patients who were reirradiated, 674 patients remained for the analysis (Figure 1). A search in the clinical database for the two largest tumor sites (tonsil and base of tongue), from 2012 to 2019, identified 890 treated cases, of which 524 (59%) were included in the registry, demonstrating the representativeness of the registry population.

The resulting cohort had a median follow-up of 10.3 months (range 5–24), and the prevalence of grade ≥ 2 xerostomia was 30%. The cohort was treated over different time periods: 16% of patients received treatment between 2012 and 2016, 36% between 2017 and 2019, and 48% between 2020 and 2024. In the earliest time period, the submandibular glands were not systematically delineated in clinical practice, leading to a greater proportion of patients being excluded from the analysis compared to the more recent time periods. Patient characteristics are described in Table 1 together with the corresponding values in the development cohort. A significant difference can be seen between the two cohorts for tumor site, concomitant/induction medical treatment, prescribed treatment, treatment technique, xerostomia prevalence, and mean doses to OAR. The oropharynx was the dominant tumor site in our cohort (76%), whereas the larynx (44%) was the most common site in the development cohort. The development cohort also received less concomitant medical treatment and excluded patients with induction chemotherapy. Both the parotid and the submandibular glands received higher doses in the development cohort compared to the validation cohort, with a mean-dose difference of 3.0 Gy and 1.8 Gy for the parotid and the submandibular glands, respectively. In the validation cohort, baseline data were available for only 99 patients. Among these, 83% had no xerostomia, 16% grade-1 xerostomia, and 1% had grade-2 xerostomia before radiotherapy.

**Table 1:** Patient and treatment characteristics of the current validation cohort (KUH) and the model development cohort (LIPP [23]), which was composed of two previous cohorts (CITOR and Initial validation [2]).

Patient characteristics	KUH (674)	LIPP (1145)	CITOR cohort (750 pat)	Initial validation cohort (395)	*p*
**Sex**					0.18
Male	**481 (71%)**	**850 (74%)**	560 (75%)	290 (73%)	
Female	**193 (29%)**	**295 (26%)**	190 (25%)	105 (27%)	
**Age** (mean, SD)	**63.0 (10.3)**	**63.3 (10.0)**	63 (10.3)	64 (9.4)	0.54
**Tumor site**					< 0.001
Oropharynx	**509 (76%)**	**411 (36%)**	271 (36%)	140 (35%)	
Nasopharynx	**20 (3.0%)**	**45 (3.9%)**	30 (4.0%)	15 (3.8%)	
Hypopharynx	**33 (4.9%)**	**121 (11%)**	71 (9.5%)	50 (13%)	
Larynx	**35 (5.2%)**	**502 (44%)**	334 (45%)	168 (43%)	
Oral cavity	**29 (4.3%)**	**66 (5.8%)**	44 (5.9%)	22 (5.6%)	
Other	**48 (7.1%)**	**0**	0	0	
**HPV status (only oropharynx)**					-
Positive	**466 (92%)**				
Negative	**38 (7.5%)**				
Missing	**5 (1.0%)**				
**Concomitant medical treatments^a^**	**457^b^ (68%)**	**466 (41%)**	242 + 65 (41%)	134 + 25 (40%)	< 0.001
**Induction chemotherapy**	**84^c^ (12%)**				-
**Prescribed treatment^d^**					< 0.001
51–64 Gy in 17–32 fractions	**25 (3.7%)**	**20 (1.7%)**	1 (0.1%)	19 (4.8%)	
66–70 Gy in 33–35 fractions	**593 (88%)**	**1118 (98%)**	747 (100%)	371 (94%)	
71.4–84 Gy in 34–35 fractions	**56 (8.3%)**	**7 (0.6%)**	2 (0.3%)	5 (1.3%)	
**Treatment technique**					< 0.001
VMAT	**668 (99%)**	**500 (44%)**	118 (16%)	382 (97%)	
IMRT	**-**	**553 (48%)**	546 (73%)	7 (1.8%)	
3D-CRT	**6 (0.9%)**	**92 (8.0%)**	86 (11%)	6 (1.5%)	
**Xerostomia grade ≥ 2**	**204 (30%)**	**531 (46%)**	342 (46%)	189 (48%)	< 0.001
**Median physical mean dose to OAR in Gy (IQR)**					
Parotid ipsilateral	**28.3 (22.4–37.9)**				
Parotid contralateral	**16.2 (11.0–19.6)**				
SMG ipsilateral	**65.2 (59.0–67.6)**				
SMG contralateral	**46.2 (39.6–51.7)**				
Parotid glands	**22.6 (17.6–27.4)**	**25.6 (14.4–33.8)**	28.0 (15.9–37.0)	21.2 (11.5–27.6)	-
Submandibular glands	**54.8 (46.3–59.3)**	**56.6 (42.2–62.5)**	58.9 (44.6–64.3)	51.9 (37.5–58.9)	-

SD: standard deviation; HPV: human papillomavirus; VMAT: Volumetric Modulated Arc Therapy; IMRT: Intensity Modulated Radiation Therapy; CRT: Conformal Radiation Therapy; OAR: organs at risk; IQR: Interquartile Range; SMG: submandibular.

a Medical treatment describes systemic therapy, which, for the validation cohort, was in the form of chemotherapy (51%), targeted therapy (13%), or a combination of both (3.9%).

b 48% cisplatin, 13% cetuximab, 4% both, or 3% other.

c Mostly TPF (docetaxel, cisplatin, and fluorouracil)

d Assuming 2–2.4 Gy fractions from Van den Bosh et al. [2].

The *p*-value refers to differences between the KUH and the total LIPP cohort (comparison not possible for the median OAR dose).

### Validation

**Figure 2:**
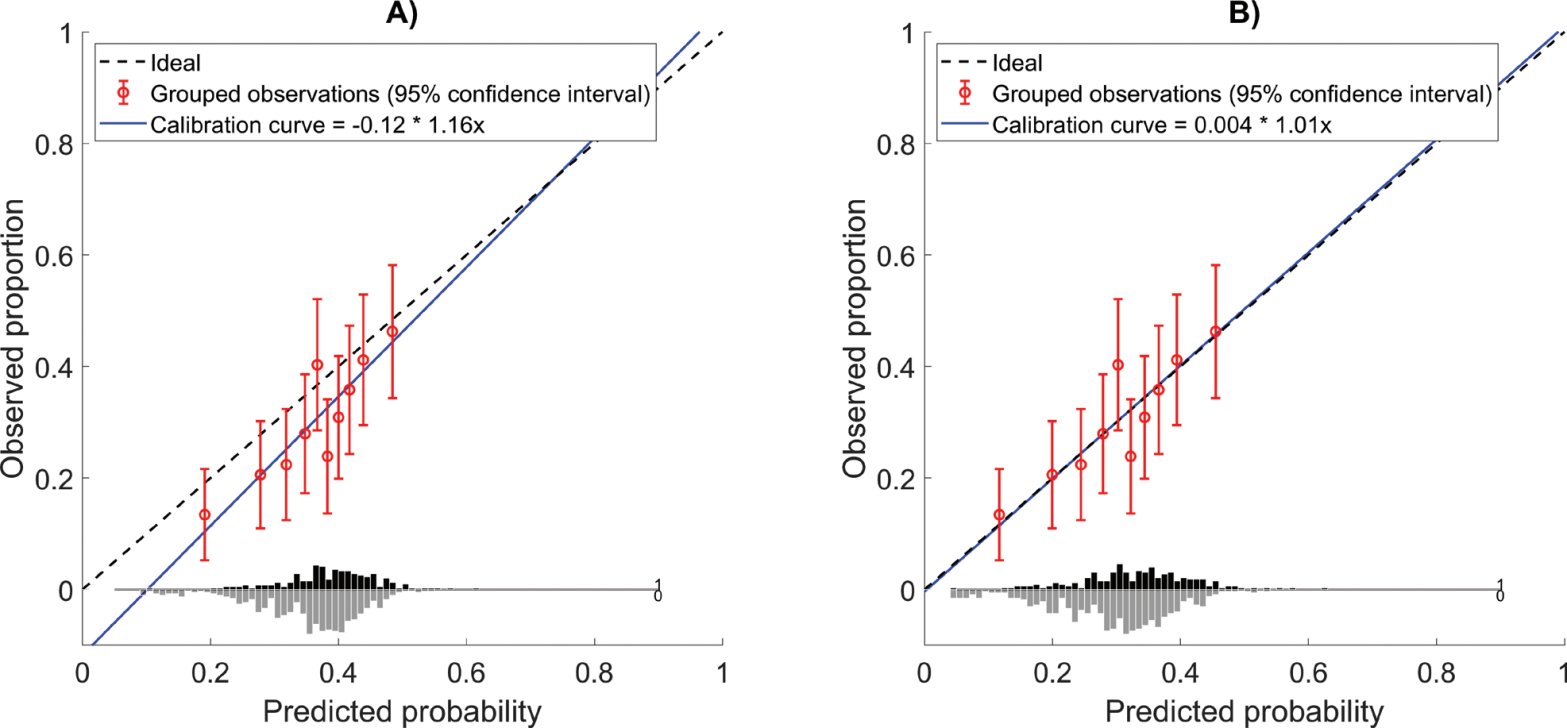
Calibration curves with the distribution of the true outcome shown at the bottom of the graph. The circles indicate the observed frequencies by deciles of predicted probability, approximately 70 patients in each bin. (A) Calibration curve before updating, with the KUH cohort in the LIPP model. (B) Calibration curve after updating by recalibration, with the KUH cohort in the recalibrated LIPP model.

The closed testing procedure indicated that the model should be recalibrated, and all the results are summarized in Table 2. Calibration-in-the-large showed a mean predicted risk of 36.2%, while the observed risk was 30.3%, indicating an overestimation of the risk by the model, which was also seen in the calibration intercept of −0.12. The calibration curve (Figure 2) demonstrated a calibration slope of 1.16, indicating a slightly stronger dose response in the validation cohort. A significant difference between predicted and observed xerostomia incidence was identified, as indicated by the HL-test (*p* = 0.014). After model recalibration, the HL-test indicated no significant lack of fit (*p* = 0.26), suggesting improved agreement between the predicted and observed outcomes.

**Table 2:** Performance of the KUH cohort in the validated model and after recalibration, together with the recalibrated performance of the LIPP model [23].

Model parameters and performance	KUH cohort in the LIPP model (validation)	KUH cohort in the recalibrated LIPP model	CITOR + Internal validation cohort in the LIPP model (development)
Intercept	−2.2951	−3.2132	−2.2951
$\sqrt{D_{mean}\left(ipsi\;Parotid\right)}+\sqrt{D_{mean}\left(contra\;Parotid\right)}$	0.0996	0.1354	0.0996
*D_mean_* (*SMG*)	0.0182	0.0247	0.0182
Log-likelihood	−404	−398	-
Calibration intercept	−0.12	0.00	0.00
Calibration slope	1.16	1.00	1.00
AUC	0.62	0.62	0.72
Hosmer-Lemeshow test *p-value*	0.014	0.26	0.23
Brier score	0.21	0.20	-

AUC: Area Under the Curve.

The model’s discrimination decreased from an AUC of 0.72 in the development cohort to 0.62 in the validation cohort. At an NTCP threshold of 30% (corresponding to our clinical dose constraints), the model showed a sensitivity of 89% and a net benefit of 0.035.

## Discussion

An external validation of the xerostomia prediction model, from the Dutch national protocol for indication of proton therapy, was conducted using a cohort of 674 patients from our local quality registry. The model required a small recalibration, which is not surprising, given the different definitions of the endpoint in the development and validation studies. However, the good fit of the recalibrated model indicates that the dose response of the parotid and the submandibular glands is well captured by the model, and that this is robust to a range of differences in cohort characteristics.

The low AUC (0.62) and Brier score (0.21) indicate weak discriminative ability, which limits the model’s accuracy when applied to an individual. Poor discrimination with good calibration may arise from a homogeneous case mix [29, 30], as in our cohort. The range of doses to the parotid and the submandibular glands and, consequently, the range of predicted probabilities were relatively narrow. The omission of the baseline symptoms also inherently reduces the discriminative ability [30]. Since the AUC reflects the performance of the model across the full range of its parameters, in practice, specific thresholds are more relevant for patient risk stratification. For our current clinical dose constraints (mean parotid dose < 25 Gy and mean submandibular gland dose < 39 Gy), the LIPP model estimates a risk of 30% (with no baseline toxicity); at this level of risk, the model shows a fair sensitivity at 89%. Although the calibration intercept and slope indicate a good calibration, the HL-test suggests a weak agreement between the predicted and observed outcomes. However, this test has been questioned due to its sensitivity to sample size and binning choices [18, 19].

A key strength of this study is that the cohort represents a real-world patient population, inspiring confidence in NTCP models for broader clinical application [17]. However, this also introduced one of the main limitations of the validation, as the timepoint for xerostomia assessment varied considerably in our cohort, ranging from 5 to 24 months post-treatment. In contrast, the original model development cohort employed a standardized assessment at 6 months. We chose the 5-to-24-month interval to avoid potential confounding from lingering acute symptoms typically seen up to 5 months, while also capturing clinically meaningful late toxicity, as xerostomia that persists up to 2 years is generally considered stable and chronic. While the primary interest was to apply the model to clinically relevant long-term xerostomia, we nonetheless explored, in a separate analysis, the effect on calibration when restricting the follow-up window to 5–12 months, to more closely mirror the original development cohort. The results remained consistent with those from the full cohort and similarly indicated a need for model recalibration, as demonstrated by a calibration intercept of –0.21 and a calibration slope of 1.48 (data provided in the Supplementary Materials). Hence, the LIPP model seemed at least equally applicable when including follow-up data up to 2 years post-radiotherapy.

The predictive accuracy for our cohort could be improved by incorporating baseline data. However, limiting the testing cohort to patients with recorded baseline data would have significantly reduced the cohort size; only 15% of the patients had baseline data available. The frequency of baseline reporting is improving in the registry, and future studies may be able to validate the impact of baseline xerostomia, provided sufficient event numbers are available for robust statistical analysis. Additionally, the absence of reported baseline xerostomia rate in the original development cohort prevents a direct comparison between the cohorts and verification of the effect size. Still, since baseline xerostomia was not present for the great majority of the patients for whom baseline data were available in our cohort, the current analysis can be considered generally applicable.

The prevalence of grade ≥ 2 xerostomia in our cohort (30%) was lower than in the LIPP cohort (46%); a difference is to be expected since xerostomia was scored according to different scales. Furthermore, differences between the cohorts likely contributed to the observed difference. For example, in our cohort, the median physical mean dose to the parotid glands was 22.6 Gy, compared to 25.6 Gy in the LIPP cohort, while the median mean dose to the submandibular glands was 54.8 Gy versus 56.6 Gy. Additionally, the higher prevalence in the LIPP cohort may be explained by differences in follow-up times, as salivary glands can recover over time [31]. The follow-up time in the validation cohort extended up to 2 years, whereas in the LIPP cohort, it was limited to 6 months. The LIPP cohort (2007–2017) and our validation cohort (2012–2024) span periods marked by considerable progress in radiotherapy and shifts in patient demographics, which can affect model performance [32]. In our clinic, notable developments include the replacement of the sequential boost technique by SIB in 2016, the introduction of RapidPlan optimization in 2019, and the reduction of the GTV-to-CTV margin in 2020 (through the 5+5-mm expansion). We are currently evaluating the impact of the latter two changes on the OAR dose and the rate of side effects, and a manuscript is in preparation. Nevertheless, despite the highlighted differences, we observed a strong correlation between predicted and observed outcomes, which supports the model’s robustness and suggests that it maintains its predictive value even when applied across differing assessment approaches and evolving clinical contexts.

The comprehensive individual toxicity risk (CITRO) profile from Van den Bosch et al. [2], including a xerostomia NTCP model, identified the oral cavity as a relevant OAR in xerostomia modeling, but only in the acute phase. Other NTCP models for xerostomia have similarly reported an association between the oral cavity dose and the xerostomia risk [12, 33, 34]. Furthermore, there is some evidence of the importance of specifically sparing the stem cell region within the salivary glands [34, 35], although this was not confirmed in a recent randomized controlled trial [13]. Proper delineation of this region requires extensive contouring guidelines and MRI for accurate identification.

De Vette et al. [36] have recently validated the CITRO profile of NTCP models from Van den Bosch et al. [2], showing a promising generalizability of the models. They had an excellent calibration close to the identity line (calibration slope ~ 1) for the xerostomia model at 6 months post-radiotherapy but showed some underestimation of the risk with an intercept of 0.43, which may be explained by the difference in side-effect assessment between the cohorts [36].

The validation of the LIPP model demonstrated good overall agreement with our data, with a further improved calibration after recalibration, supporting its applicability for predicting xerostomia in our population. As our registry expands with more baseline data, further refinements may enhance the predictive accuracy and the discrimination. While the model performed well, developing a tailored model trained on our cohort could enable the inclusion of additional predictive factors specific to our patient population, potentially enhancing the performance. Notably, differences in the xerostomia prevalence between our cohort and the LIPP cohort suggest that variations in the radiation dose distributions, follow-up duration, and grading systems may influence model performance. A locally trained model could better account for these variations, leading to an improved discrimination.

With a coverage of the registry of around 60% for the current cohort, the risk of bias needs to be considered when relying on the model in treatment planning. The missing data are expected to predominantly result from logistical challenges during data collection. Patient exclusion likely occurred without systematic bias, suggesting that the incompleteness of the dataset should have minimal impact on the results. However, the missing data will be further characterized in future work and considered in any clinical implementation.

Ultimately, our goal is to ensure validity and maximize clinical utility in the context of NTCP-based treatment planning. Depending on the planning strategy, model calibration to the population of interest may be more or less critical, that is, depending on whether decisions are made based on relative or absolute NTCP estimates. By continuously refining and externally validating predictive models, we can support more personalized and evidence-based radiotherapy strategies, improving the post-treatment quality of life for head and neck cancer patients.

## Conclusion

Despite differences in the endpoint definition and the cohort characteristics, our findings demonstrate that the LIPP model exhibits transportability and can be generalized to our real-world data after recalibration, supporting the validity of the Dutch national protocol’s model for radiation-induced xerostomia. While the recalibrated model showed good calibration, its limited discriminative ability suggests the possibility for further refinements to improve individual risk stratification.

In conclusion, our validation suggests that the recalibrated LIPP model is applicable for NTCP-based treatment planning. Further efforts should focus on integrating additional predictive factors to enhance clinical decision-making and improve post-treatment quality of life for head and neck cancer patients.

## Supplementary Material



## Data Availability

The data underlying this study are not publicly available due to ethical constraints. Access to the analysis code may be granted upon reasonable request to the first author.
