# Supplementary material for: Validated prediction of xerostomia in a real-world population: a step toward model-guided radiotherapy
Source: Acta Oncol. 2025 Aug 18;64:43462. doi: 10.2340/1651-226X.2025.43462 (PMC12371747; doi:10.2340/1651-226X.2025.43462)
Supplement: Supplementary file 1 [file AO-64-43462-s1.pdf]

Supplementary material has been published as submitted. It has not been copyedited, or typeset by Acta Oncologica

Supplementary materials

Decision curve analysis:

The clinical implementation of NTCP models presents significant challenges, raising critical questions about model calibration and its potential benefit over existing clinical standards. Specifically, it is essential to determine whether a model is calibrated well enough for clinical application and whether it provides a meaningful improvement over conventional dose constraint. Incorrect calibration can lead to systematically flawed decision-making and while perfect calibration and strong discriminatory ability improve decision-making, they do not inherently guarantee clinical utility [1]. To evaluate the clinical utility of prediction models, net benefit (NB) is frequently used within decision curve analysis (DCA), a statistical method that assesses whether a model enhances clinical decision-making [1-4]. Net benefit is defined as:

$$NB = \frac{TP}{N} - \frac{FP}{N} \cdot \frac{p}{1-p}$$

TP (True Positives): Correct identified high-risk patients (over threshold)
FP (False Positives): Patients incorrectly classifieds as high-risk
N: Total number of patients
p/(1-p): odds of the threshold (Weighting factor, where p = threshold probability)

This measure assumes that an action is taken when a predefined risk threshold is exceeded and that using the model should provide a net benefit compared to treating all or treating none. This does not strictly apply to the current scenario, as dose constraints serve as guidelines in treatment planning, but in the case of xerostomia, OAR sparing is often deprioritized in favour of maintaining target coverage.
Even so, DCA framework can still provide valuable insights even if direct clinical actions based on risk thresholds are not applicable in our study. By evaluating the net benefit across different threshold probabilities, DCA allows us to assess whether incorporating the NTCP model leads to improved decision-making compared to existing treatment approaches. It can also help identify threshold ranges where the model offers the greatest clinical value, even if strict threshold-based interventions are not implemented. A well-calibrated model cannot be harmful (Negative NB), supporting the argument that our validation of the LIPP model is sufficiently calibrated for clinical use [1]. Also, DCA enables model comparisons, illustrating whether the inclusion of additional predictive factors enhances net benefit in future research.

An additional consideration for future research is whether the NTCP model can be used for iso-prescription decisions. Figure S1 presents the DCA curve for our recalibrated model, indicating a net benefit in the 20–50% risk range. Accepting a 30% xerostomia risk yields a net benefit, suggesting the model's potential utility in iso-prescription to enhance tumour control probability (TCP). However, for patients with unfavourable anatomy where achieving

minimal target coverage necessitates exceeding a 50% xerostomia risk, the model does not confer a net benefit. In such cases, standard treatment (without NTCP-guided prescription) may be preferable.

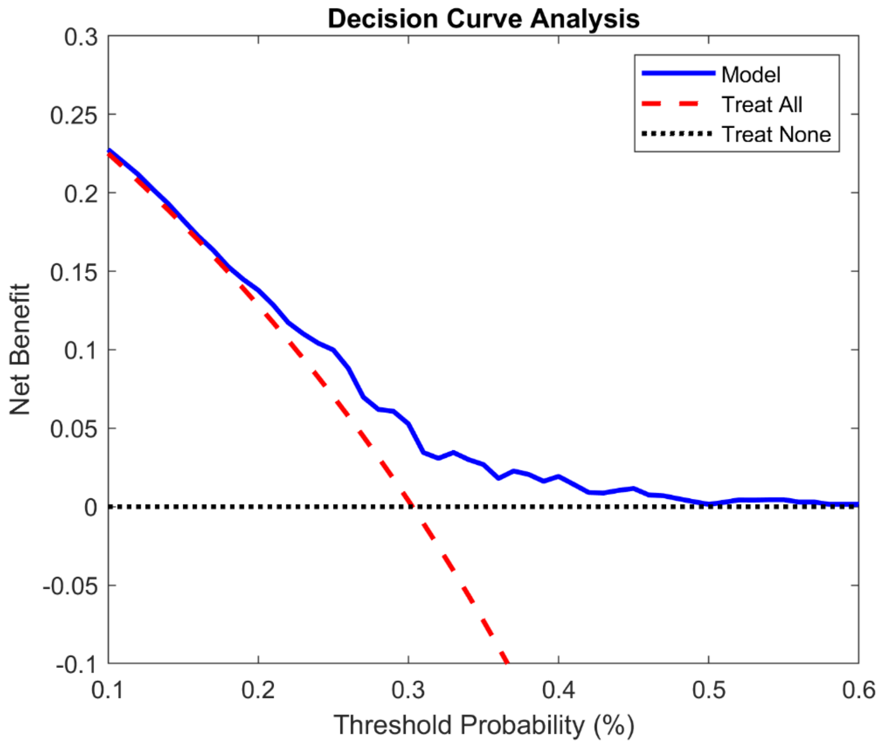

Figure S1: Decision curve analysis for the recalibrated xerostomia LIPP model in our validation cohort, illustrating the NB across the full range of possible decision thresholds. The results indicating a clinical benefit within 20-50%. The NB is compared against the "treat none" and "treat all" strategies. The treat none curve remains at zero NB, as there are no TP or FP. While "treat all" curve varies based on the threshold probability and outcome incidence, reflecting a scenario where all patients are treated—resulting in no false negatives but a high risk of overtreatment [1].

Restricted cohort results:

Restricting the follow-up window to 5-12 months led to a cohort of 454 patients with a median follow-up of 7.4 months (range 5-2). The results remained consistent with those from the full cohort and similarly indicated a need for model recalibration.

	**Restricted KUH cohort in LIPP model**	**Restricted KUH cohort in recalibrated LIPP model**
Intercept	-2.2951	-3.884
$\sqrt{D_{mean}(ipsi\ Parotid)} + \sqrt{D_{mean}(contra\ Parotid)}$	0.0996	0.1782
$D_{mean}(SMG)$	0.0182	0.0326
Log-likelihood	-273	-270
Calibration Intercept	-0.21	0.00
Calibration Slope	1.48	0.98
AUC	0.65	0.65
Hosmer-Lemeshow test *p-value*	0.18	0.63
Brier score	0.21	0.21

Table S1: Performance of the restricted KUH cohort in the validated model and after recalibration.

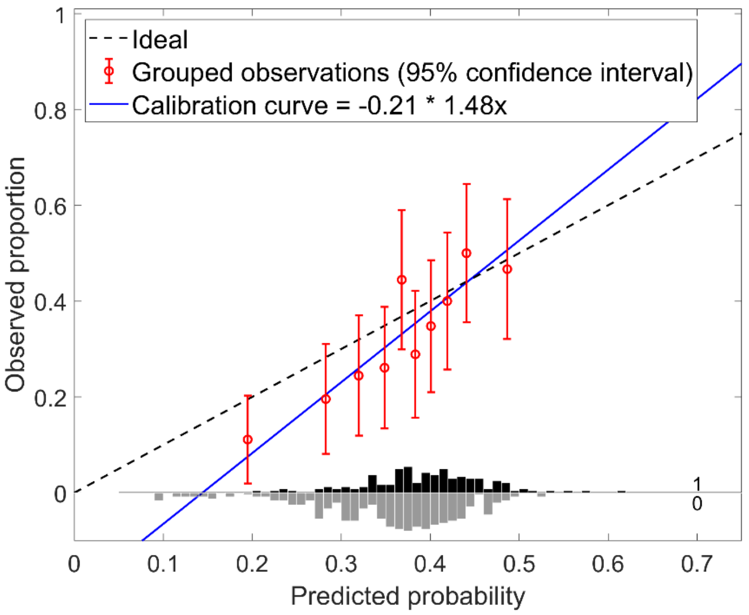

Figure S2: Calibration plot for the restricted cohort (n=454) with the distribution of the true outcome shown at the bottom of the graph. The circles indicate the observed frequencies by deciles of predicted probability, approximately 70 patients in each bin.

References

1.Steyerberg EW. Clinical Prediction Models - A Practical Approach to Development, Validation, and Updating: Springer; 2009.

2. Moons KG, Kengne AP, Grobbee DE, Royston P, Vergouwe Y, Altman DG, et al. Risk prediction models: II. External validation, model updating, and impact assessment. Heart. 2012;98(9):691-8. DOI: https://doi.org/10.1136/heartjnl-2011-301247

3. Vickers AJ, Cronin AM, Elkin EB, Gonen M. Extensions to decision curve analysis, a novel method for evaluating diagnostic tests, prediction models and molecular markers. BMC Med Inform Decis Mak. 2008;8:53. DOI: https://doi.org/10.1186/1472-6947-8-53

4. Binuya MAE, Engelhardt EG, Schats W, Schmidt MK, Steyerberg EW. Methodological guidance for the evaluation and updating of clinical prediction models: a systematic review. BMC Med Res Methodol. 2022;22(1):316. DOI: https://doi.org/10.1186/s12874-022-01801-8
